# Shaping a Plant Genetics and Molecular Biology research community in Brazil

**DOI:** 10.1590/1678-4685-GMB-2017-00050001

**Published:** 2017-04-10

**Authors:** Marie-Anne Van Sluys

**Affiliations:** 1 Departamento de Botânica, Instituto de Biociências, Universidade de São Paulo IB–USP), São Paulo, SP, Brazil

The present issue of Genetics and Molecular Biology is dedicated to explore the genetics and molecular biology of plants. Brazil has overcome food security in as little as 20 years from 1960’s to 1980’s. Moreover, it also became a major player in the world food production and a reference in tropical agriculture science. Together with classical breeding and agronomical techniques, a large group of young researchers went abroad during the 80’s to learn and bring back new plant molecular genetic tools to identify, study and modify plant gene functions of interest in Brazilian crops. Thus, a high degree of internationalization was a common feature in the origin of plant molecular genetics in Brazil. These researchers returned to Brazil and established themselves at different institutions with the task of building the so-called Plant Biotechnology Infrastructure in the early 90’s, soon after their PhD or postdoctoral period. In 2007, the first Brazilian Symposium of Plant Molecular Genetics (SBGMP - Simposio Brasileiro de Genética Molecular de Plantas) was held in Natal (RN). Since then, every two years a new Symposium occurred at different locations in Brazil: 2009, Buzios (RJ); 2011, Ilhéus, (BA); 2013, Bento Gonçalves (RS) and 2015, Iguassu Falls (PR), held within 11^th^ International Plant Molecular Biology Conference (IPMB). This special issue of Genetics and Molecular Biology celebrates the V SBGMP. It includes 11 review articles and five research articles. The inspiration to organize this special issue came from results presented at the 11th IPMB Congress held in Iguassu Falls in October 2015, which resulted from a joint initiative of researchers from Brazil and Argentina. The International Plant Molecular Biology Congresses is a triennial initiative by the Plant Research community from around the world that focuses on new discoveries on the nature of plant life at the molecular level. The 11^th^ IPMB message to the Society based on the Organizing Committee was that “Plants can help” improve quality of life and environmental conservation, mitigate climate change, and provide opportunities for a sustainable development around the globe. Leading scientists from over 25 countries of five continents met at Iguassu Falls. A total of 1,100 participant, one third being students and two-thirds scientists, met for the first time in South America. It was an amazing opportunity to reveal exciting research at the forefront of Plant Sciences, where the new generation of plant scientists vividly discussed their research projects with young and senior scientists. Five days of intensive discussion were held on 20 major subjects related to plant life. Outstanding 11 plenary talks and 55 symposia revealed first-hand knowledge and application of plant genetics and molecular biology. A glimpse on what was presented can be found in the reviews in this special issue. The research articles present studies on plant development and response to the environment, as well as phylogeny resolution using secondary structure of transcribed spacers and active endogenous viral elements. Also, reviews will update readers on a collection of plant molecular processes embracing cell cycle, plant-pathogen interaction and resistance strategies, organic and inorganic transport as means of environmental adaptation to grow, differentiate and develop to a full life cycle. Moreover, the research articles reveal how quick the area advances. Marcia Margis-Pinheiro and Marcio C. Silva-Filho diligently coordinated the revision by peers of a number of submitted manuscripts, and together with Nelson Saibo and Santiago Mora Garcia selected the 16 papers that compose this special Plant Genetics and Molecular Biology issue. We thank not only the four above but also the Brazilian Plant Genetics and Molecular Biology research community for being enthusiastic and to believe that educating and performing research in this field will make a difference.

